# Structural optimization of 3D-printed synthetic spider webs for high strength

**DOI:** 10.1038/ncomms8038

**Published:** 2015-05-15

**Authors:** Zhao Qin, Brett G. Compton, Jennifer A. Lewis, Markus J. Buehler

**Affiliations:** 1 Laboratory for Atomistic and Molecular Mechanics (LAMM), Department of Civil and Environmental Engineering, Massachusetts Institute of Technology, 77 Massachusetts Avenue, Room 1-290, Cambridge, Massachusetts 02139, USA; 2 Center for Computational Engineering, Massachusetts Institute of Technology, 77 Massachusetts Avenue, Cambridge, Massachusetts 02139, USA; 3 School of Engineering and Applied Sciences, Harvard University, 29 Oxford Street, Cambridge, Massachusetts 02138, USA; 4 Wyss Institute for Biologically Inspired Engineering, Harvard University, 60 Oxford Street, Cambridge, Massachusetts 02138, USA

## Abstract

Spiders spin intricate webs that serve as sophisticated prey-trapping architectures that simultaneously exhibit high strength, elasticity and graceful failure. To determine how web mechanics are controlled by their topological design and material distribution, here we create spider-web mimics composed of elastomeric filaments. Specifically, computational modelling and microscale 3D printing are combined to investigate the mechanical response of elastomeric webs under multiple loading conditions. We find the existence of an asymptotic prey size that leads to a saturated web strength. We identify pathways to design elastomeric material structures with maximum strength, low density and adaptability. We show that the loading type dictates the optimal material distribution, that is, a homogeneous distribution is better for localized loading, while stronger radial threads with weaker spiral threads is better for distributed loading. Our observations reveal that the material distribution within spider webs is dictated by the loading condition, shedding light on their observed architectural variations.

## Abstract (web summary)

Spider webs have some intriguing mechanical properties, but understanding of the properties is limited to individual silk fibres. Here, the authors create mimics of spider web using 3D techniques, which enables them to acquire knowledge of mechanical strength of the entire synthetic web.

## Graphical abstract

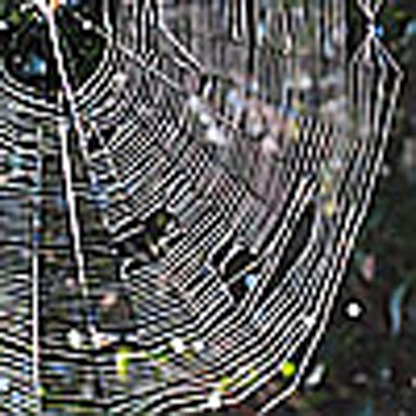

Spider webs are sophisticated architectures that exhibit exceptional mechanical properties, which are simultaneously optimized for multiple functions such as catching prey, sensing vibration and protecting offspring^1^,^2^,^3^,^4^,^5^,^6^,^7^. Of specific interest, orb webs are constructed by orb-weaving spiders by depositing protein-based silk materials through their spinnerets^6^. They are mainly composed of structural radial threads and sticky spiral threads^1^,^4^,^8^,^9^ (Fig. 1a,b). Because the energetic cost to spiders to produce silk is high, their webs are typically composed of extremely thin threads of a few micrometers in diameter, which are strong enough to sustain their functions for significant periods of time^10^,^11^. Interestingly, the overall size of different orb webs varies from centimetres to meters, while the ratio of spiral to radial thread diameter varies from ∼0.1 to 1 (refs 3, 5). Concomitantly, the extensibility and elastic modulus of the spiral and radial threads can differ by 1 and 3 orders of magnitude, respectively^3^. To understand how particular web architectures arise, we must consider the competition between minimizing energy costs to the spider and maximizing mechanical function and longevity^2^. The entire web must be studied as an integrated system, including the complex interplay between its topology, geometric parameters (for example, web size, ratio of thread diameters, thread lengths and so on) and the mechanical properties of the individual threads comprising the web.

Most assessments of the mechanics of spider webs are limited to the material property of individual silk fibres^10^,^12^. Indeed, finding suitable natural samples for experimental testing of entire webs in a repeatable way is effectively impossible. Emerging microscale three-dimensional (3D) printing techniques enable a pathway to directly fabricate and test synthetic web structures by design (Fig. 1c) to gain insight into the function of natural webs in a systematic and repeatable manner^13^,^14^,^15^,^16^,^17^ (Fig. 1d–f). 3D printing offers unique advantages over traditional fabrication routes (for example, casting or weaving) in that the geometry can be easily altered without requiring fabrication of new moulds. For example, reactive, resin-based materials can be patterned into complex web structures that would be cumbersome, if not impossible, with existing textile methods. Moreover, the computer-aided design files used to define the 3D-printed structures can directly replicate results of computational modelling and optimization to enable a tightly coupled approach for studying web mechanics. While most synthetic polymers do not have strength equivalent to natural silk, they can be used to fabricate useful web mimics that deform in a qualitatively similar way as natural webs under various loading conditions, and are therefore useful for validating the computational model and demonstrating the existence of optimal design.

Here, we report the first systematic investigation of the mechanical behaviour of synthetic, elastomeric webs as a function of their key architectural features. Specifically, we combine coarse-grained numerical models with experimental tests on printed web structures to investigate their mechanical response to localized and distributed loading. While we choose to focus on quasi-static loading conditions on webs constructed from a single elastomeric material (polydimethylsiloxane (PDMS)), our approach can be readily extended to different materials, architectures and combinations thereof^18^,^19^,^20^. We find that the web strength depends on the failure load of the radial and spiral threads, which can be controlled by varying the thread diameters. We also find that the failure mode and optimal strength of a web structure is determined by the both the nature of the loading condition and the ratio of radial and spiral thread diameters. For the case of localized loading, more uniform thread diameters (and hence equivalent failure loads) are required for optimized strength. By contrast, distributed loads require thicker radial threads (hence higher failure loads) relative to the spiral threads. Our observations reveal that the web strength depends on the material distribution, offering insight into the observations of the thickness ratio within natural spider webs. Our approach provides efficient tools to quickly realize complex elastomeric web structures with optimal mechanical functions and low density to meet critical requests of many engineering applications.

## Results

### Local failure and stiffness distribution in synthetic webs

**Figure 1:**
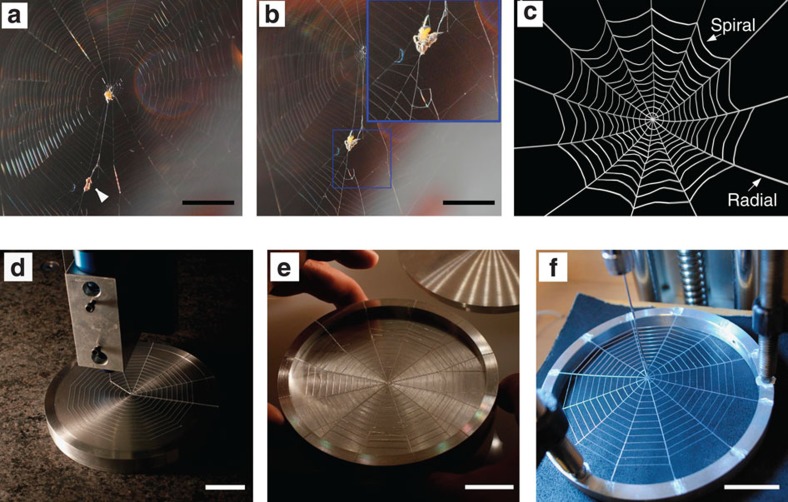
From natural to synthetic spider webs. (**a**) Photograph of a spider web, spider and small prey. The prey (indicated by an arrow) is trapped by spiral threads at the periphery of the spider web and the spider is at the centre of the web. (**b**) The spider moves from the web centre to approach the prey. Despite the flaw, the web is still able to support the weight of the spider. Scale bars in **a**,**b**: 10 mm. (**c**) Schematic figure of the computational model of an orb web under relaxation. The web is composed of radial threads and spiral threads. (**d**) The radial and spiral threads of a web are printed by a 3D printer on top of the aluminium frame and the substrate. (**e**) The frame and the web are taken off from the substrate after curing. (**f**) The frame is fixed and the thread in the web is stretched by a tensile testing machine. Scale bars in **d**–**f**: 25 mm.

**Figure 2:**
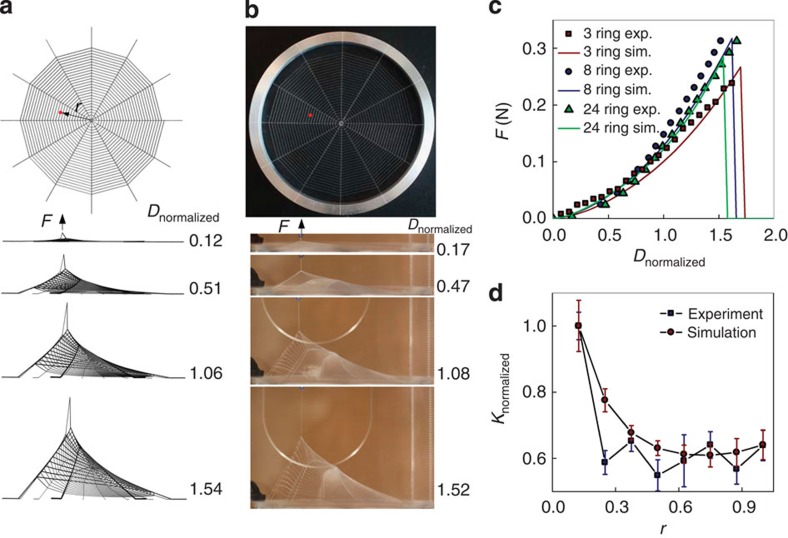
Mechanical response of synthetic webs under point loading. (**a**) Simulation snapshots of the deformation of a web structure under a point stretching force *F* applied at the middle of a spiral thread (of 24 rings of spiral threads) with distance *r*=0.35*R* from the centre of the web, where *R*=50.8 mm is the radius of the inner circle of the frame. The periphery of all the radial threads is fixed from displacement. For the sake of simplicity, we use a single rigid rod as a representative shape of prey to deform the web. The displacement of the point under loading (*D*) is normalized by *R* (*D*_normalized_=*D*/*R*). (**b**) Experiment snapshots with the same force and boundary conditions applied to the web made of PDMS via 3D printing with spiral thread diameter of *d*_s_=180 μm and radial thread diameter of *d*_r_=258 μm, identical to what is used in the numerical model. (**c**) The simulation (sim.) and experimental (exp.) force–displacement curves of three webs with different number of rings of spiral threads under loading. (**d**) Mapping the local stiffness (*K*) of a web (of eight rings) at small deformation both in simulation and experiment. This is obtained by deforming the middle point of several spiral threads and measuring the slope of the force–displacement curve at *D*_normalized_=0.1, as a function of the distance from the web centre to the loading point. The local stiffness is normalized by that of the innermost spiral thread.

The PDMS web structures are printed onto flat substrates in a circular support frame (Fig. 1d–f) using a microscale 3D printing technique, known as direct ink writing^13^,^20^. This technique enables printing cylindrical threads of uniform diameter (see Supplementary Fig. 1d, with variations in their cross-sectional area <10%) and with perfectly connected junctions yielding an integrated web structure with complex geometry. The printed web geometry mimics that of natural orb webs (Fig. 1a–c), as the radial threads mainly connect to web's anchorage (that is, at tree, floor and corner) to support the overall structure of the web, and the spiral threads are coated with aqueous glue, making them sticky for catching prey^5^,^8^,^9^. According to their different functions, we fix the ends of the radial threads to the frame and apply a point force to deform the web at the middle of a spiral thread span, applying the identical conditions in both the simulations and experiments (Fig. 2a,b). We apply quasi-static loading throughout the study by controlling the displacement of the loading point that moves in a constant small velocity to minimize the effect of any rate dependence of the material (see Methods for the detail of the tensile test). The constitutive law for the numerical model is calibrated to uniaxial tensile tests on single PDMS filaments, which results in accurate reproduction of the mechanical response of the printed web structures (Fig. 2a,b, see Methods for details). Noting that the spiral threads become nearly fully aligned with the loading direction prior to failure (with an angle <10°) because the thread under loading reaches 4.5 times of its initial length and the two points connecting to the rest of the web structure get closer under large deformation, we approximate the tensile failure stress by taking the peak force divided by twice that of the cross-sectional area of spiral thread (6.2±0.4 MPa and 6.1±0.2 MPa for the simulations and experiments shown in Fig. 2c, respectively), which approaches that of the PDMS ink used to print the thread (6.8 MPa^21^, see Methods for detail).

We change the web geometry by altering the number of spiral threads (from 3 rings to 24 rings as shown in the Supplementary Fig. 1) and repeat the loading test (Fig. 2c). We note that although the loading point is specified at the middle of a spiral thread and close to the distance 0.35*R* from the centre of the web, the actual loading location varies slightly for different web geometries. However, the simulation and experiment for each web structure are loaded in the exactly same way. We find good agreement between simulations and experiments in that rupture always happens at the loading point. This result agrees with our *in situ* observation that small prey always causes localized failure (Fig. 1a,b). We change the loading point and measure the stiffness (at a fixed displacement of 10% the radius of web) to generate a stiffness map of the web, as summarized in Fig. 2d. The good agreement between simulation and experiment shows that the stiffness of the innermost spiral threads is larger than that of the outermost spiral threads by 54%. Previous measurement of the thickness distribution in multiple natural spider webs has shown that the outermost spiral threads are larger than innermost threads by 30% (ref. 3). This distribution likely serves to make the local stiffness across the web more homogenous, since the deformation mainly arises from the single spiral thread under point loading and slightly thicker threads lead to a smaller deformation. Hence, the local stiffness for the outermost spiral threads is compensated allowing for smoother movement of the spider on the web.

### Asymptotic size effect on web strength

**Figure 3:**
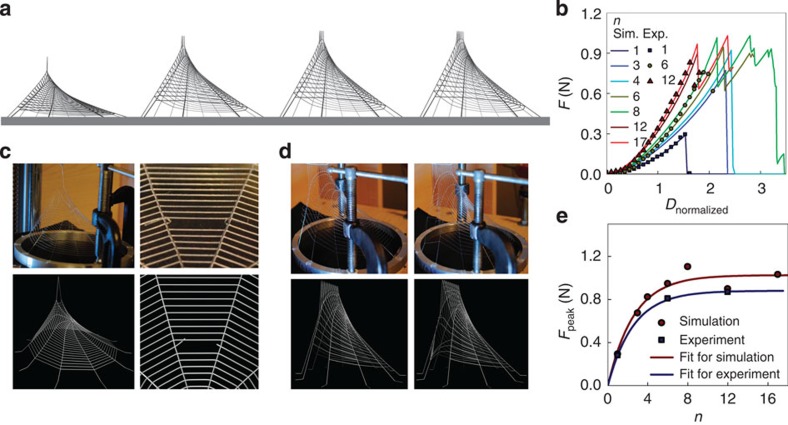
Mechanical response of synthetic webs as a function of loading size. (**a**) Simulation snapshots of the deformation of a web (of 24 rings) right before failure that is caused by different number of spiral threads under loading (*n*). (**b**) *F*–*D*_normalized_ curves of the same web under different loading conditions for different *n*. Curves are obtained both from simulation and experiment. (**c**) Comparison of the close-up of web deformation under loading with *n*=1 *in silico* (bottom) and for the experiment (top), before (left) and after (right) failure. The stretching force only breaks the spiral thread under loading. (**d**) Comparison of the close-up of web deformation under loading with *n*=12 *in silico* (bottom) and in the experiment (top), before (left) and after (right) failure. The stretching force breaks the radial threads connecting the spiral threads under loading. (**e**) Comparison of peak force (*F*_peak_) obtained from every *F*–*D*_normalized_ curve as a function of *n* for simulation and experiment. Each of those results is exponentially fitted according to equation (1), resulting in the function *F*_peak_=1.0[1–exp(−*n*/2.6)] (N) for simulation and *F*_peak_=0.9[1–exp(−*n*/2.5)] (N) for experiment result.

We increase the number of spiral threads (*n*) involved in deformation to investigate how the prey size (relative to the spiral thread spacing) affects the strength that the web can provide during deformation, as shown in Fig. 3a. A single point loading accounts for small prey such as fleas and mosquitoes, and a load case involving multiple neighbouring points accounts for large prey such as beetles and even birds^5^. Force–displacement curves (Fig. 3b) of those tests show that for *n*<6, the structure ruptures at the peak force (*F*_peak_), creating the local failure at the loading point (Fig. 3c). However, for *n*≥6, the peak force point is followed by a bumpy region that accounts for the incremental failure propagating in the radial threads, as shown in Fig. 3d. Those two different failure mechanisms are governed by the competing strength of the spiral threads and that of the radial threads in tension. The peak force, *F*_peak_, is plotted in Fig. 3e as a function of *n.* It can be seen that *F*_peak_ increases with *n,* but converges at an asymptotic level. The saturation of the force peak is caused by the transition of the failure model from a localized form (shown in Fig. 3c) by breaking the spiral threads to the diffused form (shown in Fig. 3d) by breaking the radial threads. The competition between spiral and radial threads before failure is analogous to a shear-lag model^22^,^23^, as the spiral threads are at different locations subject to different strains under loading. *F*_peak_ nonlinearly increases with the increasing number of spiral threads under loading but its plateau value is limited to the strength of the radial thread. Further increasing *n* will always break the radial threads instead of improving the strength, while the location of the saturation point should depend on the ratio of the cross-section area between the radial and spiral threads. Considering that the deformation exponentially decays from the loading edge, we use the equation:


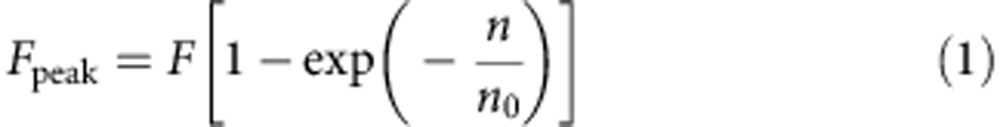


to fit the simulation and experimental results as shown in Fig. 3e, where *F* is the strength of the radial threads that connect to the spiral threads under loading and *n*_0_, which depend on the ratio of the cross-section area between the radial and spiral threads, gives the number of spiral threads above which the plateau strength is achieved. Good agreement is found between the respective *n*_0_ values obtained in simulation (*n*_0_=2.6±0.4) and experiment (*n*_0_=2.5±0.1), both of which show that 2–3 spiral threads for the current web structure (24 rings) are most efficient for *F*_peak_. Our results suggest that simply increasing the number of threads in loading (for example, by making the web denser) does not always linearly increase the web strength. These data agree with *in situ* observations that most prey captured by a given spider web are both smaller than the spider length and several times that of the spiral thread spacing^11^. Among those small prey, the larger ones are of higher value because of the large energetic return from such prey^11^,^24^, which agrees with the result that *F*_peak_ almost linearly increases with small *n* (≤*n*_0_) as revealed in Fig. 3e. This result connects to another observation that is identified across diverse species: large spiders spin better silk with both relatively higher quality and larger thickness and use it more sparsely in web construction^25^. Hence, the material distribution in web appears to be optimized for improved strength for trapping potential, which cannot always be reached by simply adding more spiral threads to the web structure.

### Spider web with scalable mechanics

**Figure 4:**
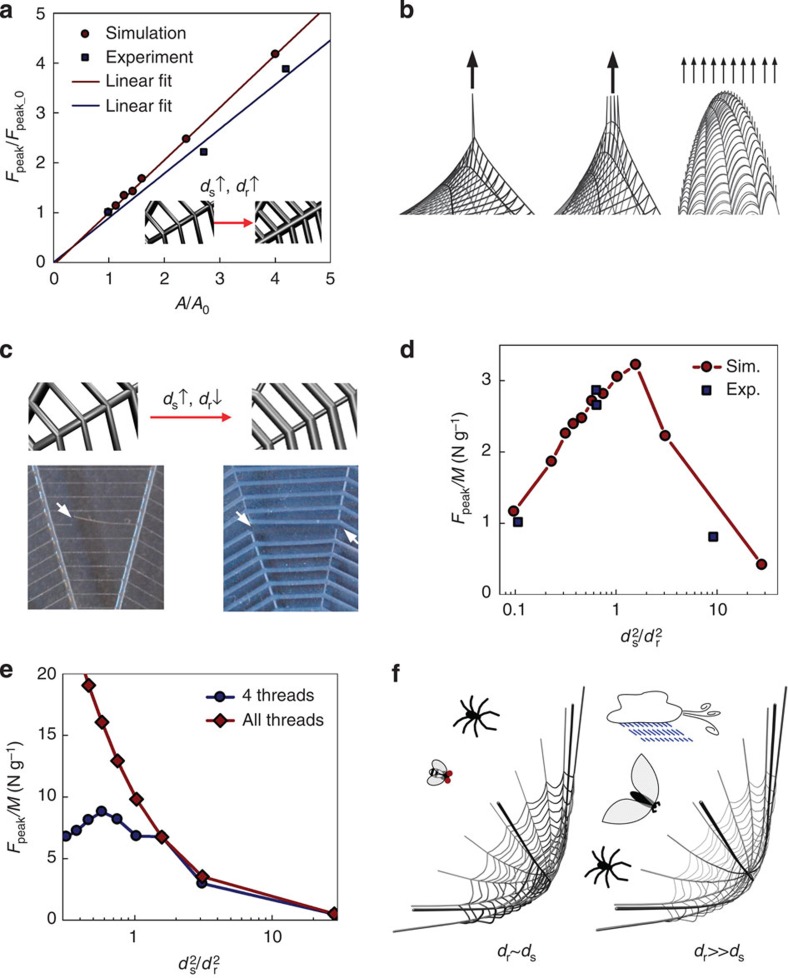
Material distribution effects on web strength. (**a**) Effect of homogeneously increasing thread diameters on PDMS web strength. We take the initial structure of *d*_s_0_=200 μm and *d*_r_0_=250 μm and keep *d*_s_/*d*_r_=0.8 constant for different models with increasing diameters for both threads. The peak force is normalized by the peak force of the initial web structure (*F*_peak_0_=0.3 N). Linear fitting gives *F*_peak_/*F*_peak_0_=(1.03±0.01)*A*/*A*_0_ for simulation and *F*_peak_/*F*_peak_0_=(0.89±0.12)*A*/*A*_0_ for experiment. (**b**) Close-up of the simulation snapshots of applying point force (*n*=1) (left), force involves four spiral threads (*n*=4) (middle), and homogenously distributed force (force on each section of each thread is proportional to its cross-section area) to the web (right). For the third case, considering that a web usually does not subject to wind and rain loading normal to the web surface, the web is initially tilted at an angle of *ϕ*=26.6° with the distributed force. (**c**) Different failure modes for webs with *d*_s_^2^/*d*_r_^2^ changing from 0.1 to 10 for point loading. The web with *d*_s_^2^/*d*_r_^2^=0.1 ruptures at the loading point while the web having *d*_s_^2^/*d*_r_^2^=10 ruptures in the radial threads away from loading. (**d**) Comparison between simulation and experiment of *F*_peak_/*M* as a function of *d*_s_^2^/*d*_r_^2^. The peak value of 3.2 N g^−1^ is reached at *d*_s_/*d*_r_=1.26. (**e**) Simulation result of *F*_peak_/*M* for the case *n*=4 and homogenously distributed force. Maximum *F*_peak_/*M*=8.8 N g^−1^ is reached at *d*_s_/*d*_r_=0.76. No peak value is identified for homogenously distributed loading, but *F*_peak_/*M* increases for decreasing *d*_s_/*d*_r_. At *d*_s_/*d*_r_→0, the maximum *F*_peak_/*M* is estimated as 140 N g^−1^ for the strength of the 12 radial threads. (**f**) Schematics of the optimized spider web in different conditions. *d*_s_∼*d*_r_ is for the case of small prey and self-weight, while *d*_r_*>>d*_s_ is necessary for large prey and rain and wind that can cause homogenously distributed force.

New webs with uniformly increased thread diameters were printed and tested for strength, as shown in Fig. 4a. The ratio of *d*_s_ (that is, the diameter of spiral threads) and *d*_r_ (that is, the diameter of radial threads) is held constant and we increase both of their cross-sectional area (by *A/A*_0_). We apply the same loading method as shown in Fig. 2a and record the peak force as a function of the scaling factor, *A/A*_0_. The normalized peak force is summarized in Fig. 4a. By scaling up the diameter of all threads, we can linearly increase the strength of the web. The slopes obtained by linear fitting shows good agreement between simulation (1.03±0.01) and experiments (0.89±0.12). These values are close to 1, suggesting that by homogeneously adding mass to the web, the strength increment of the web is the same magnitude as that of each individual thread. Therefore, the unit mass strength (*F*_peak_/*M* as *M* is the total mass of the web) is constant for a given ratio, *d*_s_/*d*_r_. It explains why thicker threads are generally necessary for larger spiders^3^, since silk materials already possess highly optimized mechanical properties (at least for the radial silk)^2^.

### Optimizing the synthetic web for multiple functions

We compute *F*_peak_/*M* of many PDMS webs with different *d*_s_/*d*_r_. *F*_peak_/*M* reflects both energy cost (proportional to *M*) to the spider for building the web and longevity and effectiveness that depend on *F*_peak_. Without loss of generality, we apply three different loading methods as shown in Fig. 4b and we measure *F*_peak_ for each loading type. We note that for single point loading and small *d*_s_/*d*_r_=0.33, the failure takes place at the spiral thread under loading, while for large *d*_s_/*d*_r_=3.1 the initial failure occurs in the radial threads connecting the spiral thread under loading, as shown in Fig. 4c. We find that *F*_peak_/*M* of the web reaches a maximum value of 3.2 N g^−1^ at *d*_s_/*d*_r_=1.26 (Fig. 4d). We find the exact same trend by performing tensile experiments on PDMS webs composed of different *d*_s_/*d*_r_ and recording their strength (Fig. 4d). For loading on four spiral threads, our computational results demonstrate that the maximum *F*_peak_/*M*=8.8 N g^−1^ can be obtained at *d*_s_/*d*_r_=0.76 (Fig. 4e). We also find that *F*_peak_/*M* keeps increasing for smaller *d*_s_/*d*_r_ for homogeneous loading on all the threads, as shown in Fig. 4e. This important finding reveals that the specific loading condition dictated by the mechanical function of the web affects how web strength can be optimized through material distribution. Moreover, the ratio of *d*_s_ and *d*_r_ in a real spider web may not be optimized for solely one particular loading type, but resides at some intermediate value, because the web needs to satisfy more than one simple mechanical function.

## Discussion

The results of the web optimization provide insight into understanding how natural spider webs can range over 2 orders of magnitude in size, yet achieve similar mechanical functions. Small orb webs, made daily by garden spiders, are mainly built for catching small prey and supporting the weight of the spider^3^. Their webs are built for point loading, for which *d*_s_≈*d*_r_, as revealed by our study, provides the maximum web strength. This finding agrees with measured diameters of spiral (2∼4.8 μm) and radial threads (1∼6 μm) of many garden spiders' webs (Fig. 4f)^2^,^3^,^26^. By contrast, giant orb webs are made by spiders in rainforest areas. For example, the spider webs made by *Caerostris darwini*, found in Madagascar, can extend up to tens of meters, are made to catch much larger prey and withstand frequent rain and typhoon winds and they are usually repaired for several days before replacement^5^. Under these more distributed loading conditions, web designs in which *d*_s_<<*d*_r_ yield higher strength and allow localized failures to be repaired. This finding is in good agreement with thread diameters measured for *C. darwini* webs, which exhibit rather small values of *d*_s_/*d*_r_∼0.1 (ref. 5; Fig. 4f).

In nature, web building is an energetically costly process, making it critical that the entire web is strong enough to meet different loading demands^11^. By creating and modelling synthetic elastomeric webs inspired by nature, we have shown that the mechanical strength of the entire synthetic web is not solely due to the strength of individual threads, but as well to the web architecture. By keeping the total web mass and web topology constant to ensure that the fixed and loading boundary conditions are consistent, we optimized the web strength solely by adjusting the material distribution. Indeed, by controlling the ratio of the diameter of spiral and radial threads (and hence the failure load), the mechanical response of the synthetic web under loading can be systematically tuned and optimized for the given amount of material. We learn this lesson from synthetic webs, but the knowledge gained is useful for understanding how spiders utilize a limited amount of silk proteins to create optimal prey-trapping architectures. Harnessing this insight, we can now create synthetic analogues that possess high strength and low density for a wide range of engineering applications, such as reinforcement of composite materials, scaffolds for biomedical applications or for space exploration. Moreover, by combining computational modelling with novel 3D printing methods, one can use the same material to reach different desired mechanical functions in a facile and precise manner.

## Methods

### Stiffness model of synthetic PDMS

We design the stress–strain curve of the PDMS material for our computational model according to the Arruda–Boyce model for rubber elasticity, and determine the parameters according to experimental results. The stress–strain relationship is given as^27^


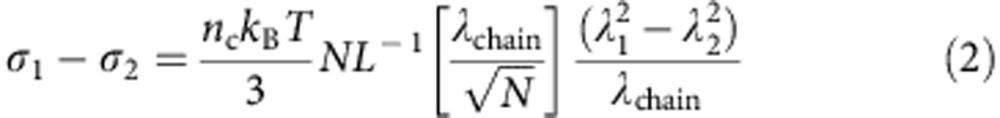


Where *σ*_1_ and *σ*_2_ are principal values of stress, *n*_c_ is the chain density, *N* is the number of rigid links of equal length for each polymer chain, *k*_B_ is the Boltzmann's constant, *T* is the temperature, *λ*_chain_ is the average chain stretch, *λ*_1_ and *λ*_2_ are principal values of stretch, and *L*^−1^(*a*) is the inverse Langevin function with Padé approximant^28^ as 
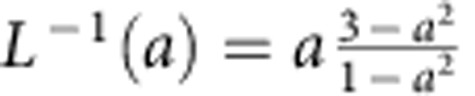
. For uniaxial tension, *σ*_2_=0, *λ*_1_=1+*ɛ*, *λ*_2_=*λ*_3_. We use the condition of incompressibility and obtain 
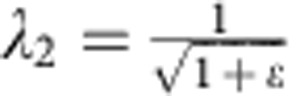
 and 
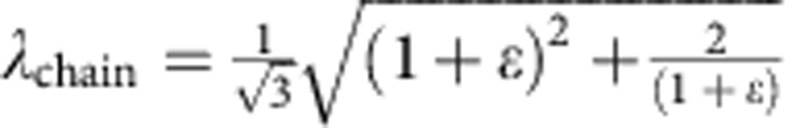
. The uniaxial engineering stress–strain relation of each individual thread is expressed as


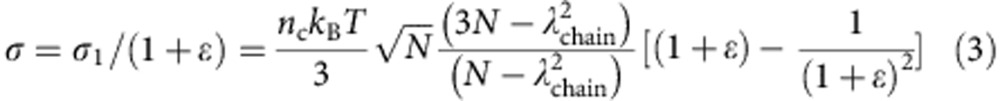


where *n*_c_=*N*_A_*ρ*/*M* as *ρ*=965 kg m^−3^ is the density of PDMS and *M* is the average molecular weight between cross-links with its numerical value between 4 and 40 kDa^29^. *M* and *N* are unknown in this equation. For the current PDMS at *T*=300 K, we know that the failure strain is *ɛ*_*b*_=355%, yielding the chain stretch as *λ*_chain_=2.655, and the corresponding stress yields 6.8 MPa^21^. From our own tensile test on the single PDMS thread, it is obtained that the stress is 1.2 MPa for *ɛ*=1, yielding the chain stretch as *λ*_chain_=1.291. By combining those two conditions, we solve the unknown parameters in this rubber model as *M*=12.68 kDa and *N*=10.2 and the constitutive law for this PDMS used in our study is


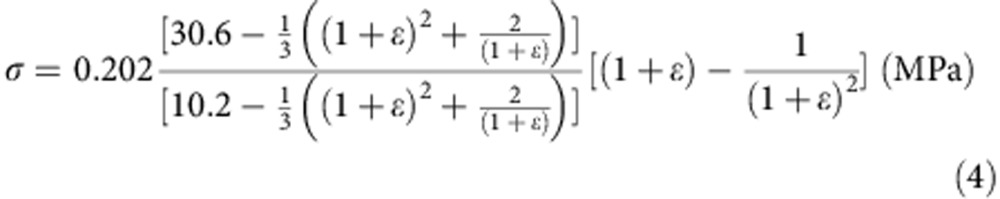


This function gives the stress–strain curve as Supplementary Fig. 1e, which shows excellent agreement with the single PDMS thread test in experiment.

### Computational model of synthetic spider web

According to equation (4), we implement the PDMS model into the LAMMPS package^30^ by defining the bond force as


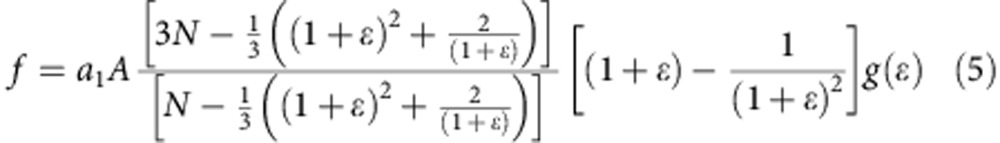


Where *A*=*πd*^2^/4 is the cross-section area of the thread as *d* is the diameter of the thread, *ɛ*=Δ*r*/*r*_0_ as *r*_0_ is the equilibrium distance between two bonded beads, and Δ*r* is the stretch of the bond and the Fermi–Dirac distribution function 
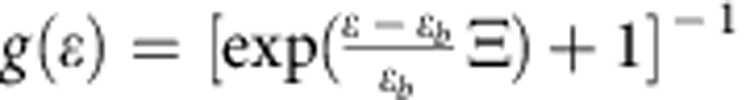
 severs the cut-off function at the ultimate strain *ɛ*_*b*_=355%, where Ξ is the smoothing factor around the rupture point. It is noted that 
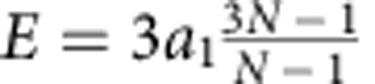
 yields the Young's modulus of the material at small deformation. The angular spring that defines the bending stiffness of each fibre is a function of the thread diameter, which is given by


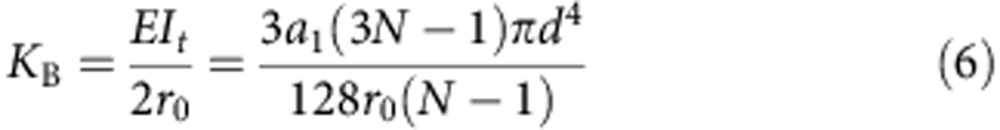


Damping effects are included in the model by considering the energy dissipation of each thread in motion caused by the viscosity of air in room temperature. Because of the small dimension and slow motion in test of every thread, we use the Stokes' law to measure the drag force by^31^


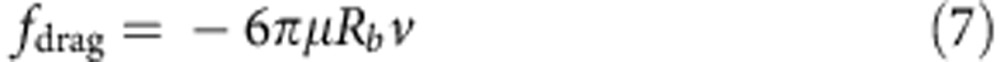


where *μ*=1.86 × 10^−5^ Pa s is the viscosity constant of air at room temperature, *R*_*b*_ is the equivalent spherical radius of the mesoscopic bead in computational model and *v* is the relative velocity of particle motion in air. The numerical values of all the parameters for the computational model are summarized in Supplementary Table 1.

### Synthetic web printing

To fabricate synthetic webs, SE 1700 PDMS from Dow Corning (Midland, MI, USA) is loaded into 3 cc, Luer–Lok syringes (Nordson EFD, Westlake, OH) and centrifuged at 3,900 r.p.m. for 10 min to remove bubbles. Loaded syringes are then placed in an HP3 high-pressure adaptor (Nordson EFD), which is mounted on an Aerotech 3-axis positioning stage (Aerotech, Inc., Pittsburgh, PA) for deposition. The PDMS ink is driven pneumatically and controlled via an Ultimus V pressure box (Nordson EFD), which interfaces with the Aerotech motion control software. Luer-lock syringe tips are used to define filament (or thread) diameter, and spiral and radial filaments are printed directly onto the two-part aluminium frame shown in Fig. 1d–f. To prevent adhesion, the central region of the frame was coated with candle maker's mold release agent. The print path for each web design is generated directly from the computer models used for numerical simulations. Printed webs are cured at 160 °C for 30 min. After curing, the webs and outer frame are gently removed from the support, as shown in Fig. 1e, resulting in free-spanning webs ready for mechanical testing.

### Tensile tests

The tensile tests are carried out for every web under ambient, quasi-static conditions with a tensile testing machine. In each of the tensile tests, the frame of the web is clamped to the substrate, a rigid hook connecting to the force transducer is set to stretch certain number of spiral threads for out-of-plane displacement. The hook system, as illustrated in Fig. 1f, is made of a steel rod that is bent into two parts of an ‘L' shape: a longer part that is glued and screwed to be rigidly connected to a force transducer and the shorter part that can be used to set hook to deform several threads. The stiffness of the hook is much larger than the PDMS web; hence, it does not significantly deform during loading to affect the displacement boundary condition. All the threads under loading have the same amount of displacement along the initially out-of-plane direction (as shown in Fig. 3d). The force transducer is connected to a rigid linear motion part which moves at a constant velocity of 29 μm s^−1^. The total force applied to the synthetic web and the total displacement of the substrate is scanned by the computer that is connected to the force transducer via the parallel port at a constant frequency of 10 Hz and saved to the log file. Each sample is kept in a protective container after printing and before performing the test.

## Additional information

**How to cite this article:** Qin, Z. *et al*. Structural optimization of 3D-printed synthetic spider webs for high strength. *Nat. Commun.* 6:7038 doi: 10.1038/ncomms8038 (2015).

## Supplementary Material

Supplementary Information Supplementary Figure 1 and Supplementary Table 1
