# Supplementary material for: Structural optimization of 3D-printed synthetic spider webs for high strength
Source: Nat Commun. 2015 May 15;6:7038. doi: 10.1038/ncomms8038 (PMC4479035; doi:10.1038/ncomms8038)
Supplement: Supplementary Information — Supplementary Figure 1 and Supplementary Table 1 [file ncomms8038-s1.pdf]

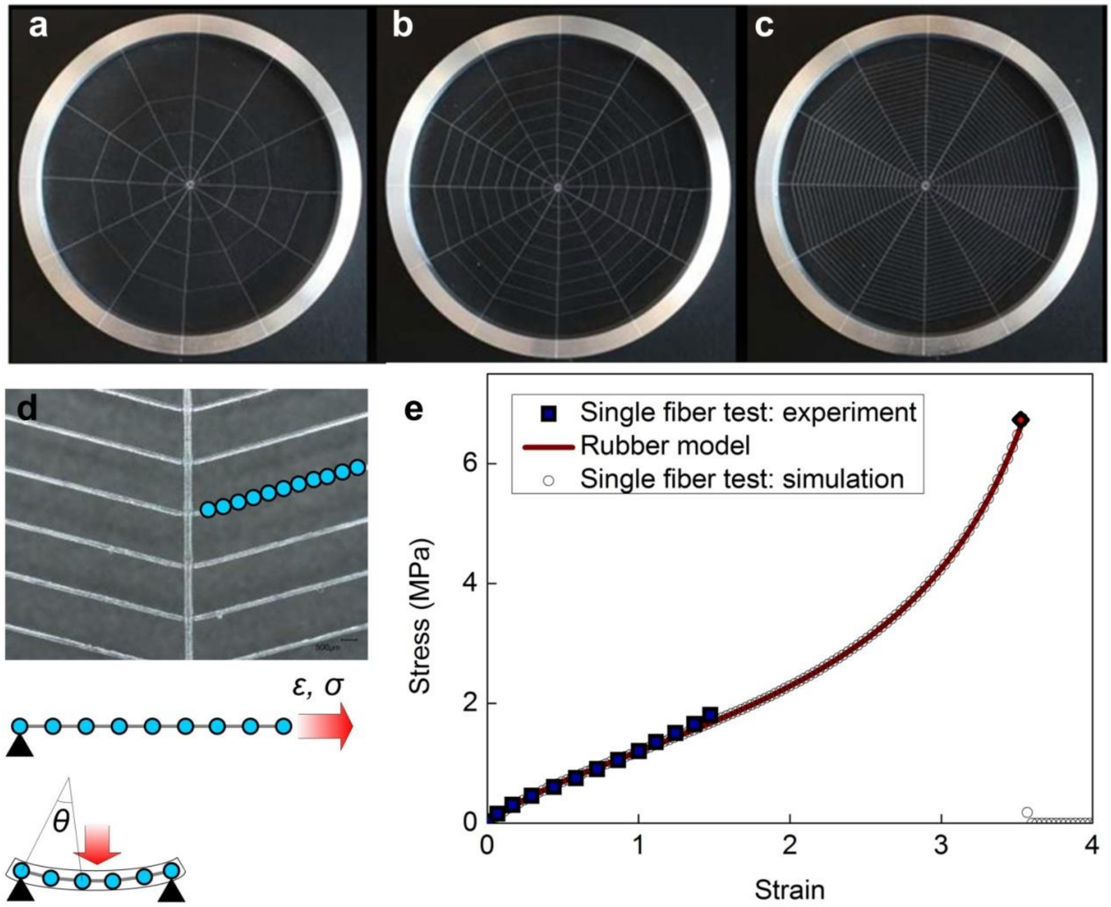

Supplementary Figure 1 | Synthetic elastomer web designs and material properties. a, the web of 3 rings of spiral threads **b,** the web of 8 rings of spiral threads **c,** the web of 24 rings of spiral threads **d,** close-up of the printed web and schematic of the bead-spring model used to model the web structure, scale bar: 500 μm **e,** comparison of the stress-strain curve obtained by uniaxial stretching of a individual PDMS given by experiment (before reaching failure, its failure stress (6.8 MPa) and failure strain (355%) are given by literature as is marked by the diamond), theoretical model and computational modeling implemented with the theoretical model.

Supplementary Table 1: The numerical values of all the parameters for the computational model.

Parameters and units	Numerical values
Stiffness constant a_1 (MPa)	0.202
Length constant of polymer N	10.2
Standard cross-section area of the spiral and radial thread A (m^2)	$\pi d_r^2/4,\ \pi d_s^2/4$
Rupture strain ε_b	355%
Equilibrium distance between two bonded beads r_0 (m)	1.4×10^{-4}
Equilibrium angle of angular springs θ_0 (in rad)	Π
Density of PDMS ρ (kg m^{-3})	965
Mass of each bead m	$\rho A r_0$
Smoothing factor Ξ	300
Drag force factor $6\pi\mu R_b$ (in N s m^{-1})	4×10^{-8}
